# The Post-office Department Suppresses Another Fraud

**Published:** 1904-11

**Authors:** 


### THE POST-OFFICE DEPARTMENT SUPPRESSES ANOTHER FRAUD.

The post-office department has issued a fraud order against the Thomas A. Edison, Jr., Chemical Company of New York, declaring the operations of this company to be fraudulent attempts to make money from the gullible public through the influence of the famous name of Edison. We have previously referred to the so-called magno-electric vitalizer, which this company puts out. It seems that two attempts were made to patent this device, which is said to consist of two plates of copper with interposed acidulated blotting paper, and the patent office refused both applications, pronouncing the apparatus inoperable and therefore not patentable. The advertisements of the company have proclaimed the marvelous invention of the young Edison, and detailed at length the remarkable effects on disease said to be secured by the use of this instrument. The senior Edison declares that his son has never shown any ability as an inventor or an electrical expert, and that the chemical company has simply given him a salary for the use of his name. The company has issued a paper called *The Magnet*, said to be edited by Thomas A. Edison, Jr. In one issue an editorial headed "Father and Son" dilates on the wonderful ability of both the Edisons and the fame which will be attached to their names in future histories. The famous inventor comes tardily, it seems to us, to the rescue of his reputation. It has been charged that he was not wholly disinterested in his son's enterprise. We trust that there is no truth in such a statement. We prefer to believe that his endorsement of the appropriate action taken by the post-office department has been preceded by serious attempts to correct his wayward son and to restrain the company from linking his name with this notorious fraud. Incidentally, it is of interest to remark that this post-office regulation ought to be an effective bar to such concerns as the one under discussion. The post-office seems to be ready to do its part; it is left for the public and the profession to bring to the notice of the authorities the many frauds which are fattening on the public through the medium of the mails.—*Journal A. M. A.*

